# Class I histone deacetylases 1, 2 and 3 are highly expressed in renal cell cancer

**DOI:** 10.1186/1471-2407-8-381

**Published:** 2008-12-19

**Authors:** Florian R Fritzsche, Wilko Weichert, Annika Röske, Volker Gekeler, Thomas Beckers, Carsten Stephan, Klaus Jung, Katharina Scholman, Carsten Denkert, Manfred Dietel, Glen Kristiansen

**Affiliations:** 1 Institute of Pathology, Charité – Universitätsmedizin, Berlin, Germany; 2 Department of Urology, Charité – Universitätsmedizin, Berlin, Germany; 3 Nycomed GmbH, Konstanz, Germany; 4 Oncotest GmbH, Freiburg, Germany; 5 Berlin Institute for Urologic Research, Berlin, Germany; 6 Institute of Surgical Pathology, UniversitätsSpital Zürich, Zurich, Switzerland

## Abstract

**Background:**

Enhanced activity of histone deacetylases (HDAC) is associated with more aggressive tumour behaviour and tumour progression in various solid tumours. The over-expression of these proteins and their known functions in malignant neoplasms has led to the development of HDAC inhibitors (HDI) as new anti-neoplastic drugs. However, little is known about HDAC expression in renal cell cancer.

**Methods:**

We investigated the expression of HDAC 1, 2 and 3 in 106 renal cell carcinomas and corresponding normal renal tissue by immunohistochemistry on tissue micro arrays and correlated expression data with clinico-pathological parameters including patient survival.

**Results:**

Almost 60% of renal cell carcinomas expressed the HDAC isoforms 1 and 2. In contrast, HDAC 3 was only detected in 13% of all renal tumours, with particular low expression rates in the clear cell subtype. HDAC 3 was significantly higher expressed in pT1/2 tumours in comparison to pT3/4 tumours. Expression of class I HDAC isoforms correlated with each other and with the proliferative activity of the tumours. We found no prognostic value of the expression of any of the HDAC isoforms in this tumour entity.

**Conclusion:**

Class I HDAC isoforms 1 and 2 are highly expressed in renal cell cancer, while HDAC 3 shows low, histology dependent expression rates. These unexpected differences in the expression patterns suggests alternative regulatory mechanisms of class I HDACs in renal cell cancer and should be taken into account when trials with isoform selective HDI are being planned. Whether HDAC expression in renal cancers is predictive of responsiveness for HDI will have to be tested in further studies.

## Background

The family of histone deacetylases (HDAC) comprises 18 isoforms which are categorized into four classes. Functionally, HDACs have been demonstrated to be involved in the deacetylation of histone tails in the nucleosomal organization units which leads to a tighter wrapping of the DNA around the histone core, which in turn results in an activation or inhibition of gene transcription [1]. In addition, HDACs influence the direct acetylation pattern of a variety of tumour relevant non-histone proteins, thus influencing their subcellular localization, interaction partners and functions [2,3]. Expression patterns of HDACs in solid human tumours have been in the focus of our group and many oncological researchers alike [4-9]. This research has been mainly triggered and promoted by the development of potent HDAC inhibitors (HDI) that have already advanced to late phase clinical trials for a broad variety of malignant human neoplasms [10,11]. An example is vorinostat, an unselective HDI, that has recently been approved for therapy of cutaneous T-cell lymphoma by the Food and Drug Administration [12]. HDI inhibit the enzymatic function of HDACs and thus change the epigenetic configuration of the tumour cells genome which influences the functions of many proteins [13,14]. Two of the most famous and best studied representatives of this group of chemotherapeutics are valproic acid (VPA) and suberoylanilide hydroxamic acid (SAHA, vorinostat). Both inhibit the function of class I and class II HDACs which has experimentally been proven to cause growth arrest, differentiation and/or apoptosis of cancer cells *in vitro *and *in vivo *[15-20]. Furthermore, HDI sensitize tumour cells for radiation induced apoptosis [21]. Surprisingly, the specific role of the different HDAC isoforms in carcinogenesis and tumour progression of renal cell cancer is not well understood.

Renal cell carcinoma (RCC) is one of the most lethal genito-urinary malignancies with about 13.000 estimated cancer related deaths in the USA in 2008 [22]. Current therapies for renal cell cancer include total nephrectomy or partial nephron-sparing surgery and chemotherapeutics like interferons or interleukins. Recently *in vitro *studies and animal experiments have shown a potential use of HDI in this tumour entity [18-20]. In this study, we analyzed expression of the class I HDAC isoforms 1–3 in a clinically well characterized patient cohort of RCC to clarify the diagnostic and/or prognostic value of these enzymes.

## Methods

### Patient characteristics

One-hundred-six patients diagnosed for renal cancer at the Institute of Pathology, Charité – Universitätsmedizin Berlin between 2003 and 2005 were enclosed in this study. The Charité University Ethics Committee has approved the study under the title 'Retrospektive Untersuchung von Gewebeproben mittels immunhistochemischer Färbung und molekularbiologischer Methoden' ('Retrospective analysis of tissue samples by immunohistochemistry and molecular biological techniques') (EA1/06/2004) on 20 September 2004. Patient age ranged between 28 and 92 years with a median of 62. Histological diagnosis was established according to the guidelines of the World Health Organization [23]. Cases were selected according to tissue availability and were not stratified for any known preoperative or prognostic factor. 84 (79.3%) patients had clear cell RCC (ccRCC), 17 (16.0%) papillary RCC and 5 (4.7%) chromophobe RCC. Twenty-three patients had systemic disease (M1, evaluated by preoperative CT-scan) at the time of diagnosis. Clinical follow-up data, as annually assessed survival time was available for all patients. The median follow-up time of all cases was 30 months, ranging from one to 47 months. Twenty-two (20.8%) patients died from renal cancer during follow up. The pT status was as follows: pT1 – 53 (50.0%), pT2 – 3 (2.8%), pT3 – 47 (44.3%) and pT4 – 3 (2.8%). Twelve patients (11.3%) had pathologically confirmed nodal metastases. Fifty (47.2%) patients had no nodal metastases (pN0). In 44 (41.5%) patients lymph nodes were not examined (pNx). Tumour grades, according to Fuhrman, were G1 – 11 (10.4%), G2 – 74 (69.8%), G3 – 17 (16.0%) and G4 – 4 (3.8%) respectively (Table 1).

**Table 1:** Expression of class I HDAC isoforms in renal cell carcinoma stratified for selected tumour parameters

	**Total**	**HDAC1 low**	**HDAC1 high**	**p-value**	**HDAC2 low**	**HDAC2 high**	**p-value**	**HDAC3 low**	**HDAC3 high**	**p-value**
**All Cases**	106 (100%)	47 (44.3%)	59 (55.7%)		46 (43.4%)	60 (56.6%)		92 (86.8%)	14 (13.2%)	
**Age ≤ 61**	49 (46.2%)	23 (46.9%)	26 (53.1%)	0.696^+^	19 (38.8%)	30 (61.2%)	0.434^+^	42 (85.7%)	7 (14.3%)	0.781^+^
**Age >61**	57 (53.8%)	24 (42.1%)	33 (57.9%)		27 (47.4%)	30 (52.6%)		50 (87.7%)	7 (12.3%)	
**pT1**	53 (50.0%)	20 (37.7%)	33 (62.3%)	0.088*	19 (35.8%)	34 (64.2)	0.088*	43 (81.1%)	10 (18.9)	0.052*
**pT2**	3 (2.8%)	0 (0.0%)	3 (100.0%)		0 (0.0%)	3 (100.0%)		2 (66.7%)	1 (33.3%)	
**pT3**	47 (44.4%)	25 (53.2%)	22 (46.8%)		26 (55.3%)	21 (44.7%)		44 (93.6%)	3 (6.4%)	
**pT4**	3 (2.8%)	2 (66.7%)	1 (33.3%)		1 (33.3%)	2 (66.7%)		3 (100.0%)	0 (0.0%)	
**Grading G1**	11 (10.4%)	6 (54.5%)	5 (45.5%)	0.596*	1 (9.1%)	10 (90.9%)	0.207*	10 (90.9%)	1 (9.1%)	0.602*
**Grading G2**	74 (69.8%)	33 (44.6%)	41 (55.4%)		42 (56.8%)	32 (43.2%)		64 (86.5%)	10 (13.5%)	
**Grading G3**	17 (16.0%)	6 (35.3%)	11 (64.7%)		3 (17.6%)	14 (82.4%)		15 (88.2%)	2 (11.8%)	
**Grading G4**	4 (3.8%)	2 (50.0%)	2 (50.0%)		0 (0.0%)	4 (100.0%)		3 (75.0%)	1 (25.0%)	
**pN0**^#^	50 (47.2%)	27 (54.0%)	23 (46.0%)	0.335^+^	26 (52.0%)	24 (48.0%)	0.339^+^	44 (88.0%)	6 (12.0%)	1.000^+^
**pN1/2**	12 (11.3%)	4 (33.3%)	8 (66.7%)		4 (33.3%)	8 (66.7%)		11 (91.7%)	1 (8.3%)	
**M0**	83 (78.3%)	37 (44.6%)	24 (55.4%)	0.559^+^	37 (44.6%)	46 (55.4%)	0.412^+^	72 (86.7%)	11 (13.3%)	.642^+^
**M1**	23 (21.7%)	10 (43.5%)	13 (56.5%)		9 (39.1%)	14 (60.9)		20 (87.0%)	3 (13.0%)	
**HDAC1 *low***	47 (44.3%)	-	-	-	31 (66.0%)	16 (34.0%)	<0.001^+^	43 (91.5%)	4 (8.5%)	0.255^+^
**HDAC1 *high***	59 (55.7%)	-	-		15 (25.4%)	44 (74.6%)		49 (83.1%)	10 (16.9%)	
**HDAC2 *low***	46 (43.4%)	31 (67.4%)	15 (32.6%)	<0.001^+^	-	-	-	45 (97.8%)	1 (2.2%)	0.003^+^
**HDAC2 *high***	60 (56.6%)	16 (26.7%)	44 (73.3%)		-	-		47 (78.3%)	13 (21.7%)	
**HDAC3 *low***	92 (86.8%)	43 (46.7%)	49 (53.3%)	0.255^+^	45 (48.9%)	47 (51.1%)	0.003^+^	-	-	-
**HDAC3 *high***	14 (13.2%)	4 (28.6%)	10 (71.4%)		1 (7.1%)	13 (92.9%)		-	-	
**Ki-67 index ≤ 10%**	70 (66.0%)	38 (54.3%)	32 (45.7%)	0.007^+^	37 (52.9%)	33 (47.1%)	0.007^+^	64 (91.4%)	6 (8.6%)	0.068^+^
**Ki-67 index > 10%**	36 (34.0%)	9 (25.0%)	27 (75.0%)		9 (25.0%)	27 (75.0%)		28 (77.8%)	8 (22.2%)	

^+ ^Fisher's exact test, * Chi-square test for trends, ^# ^44 cases (41.5%) were pNx

### Tissue Micro Array construction

A tissue micro array (TMA) was constructed as previously described [24]. Suitable areas for tissue retrieval were marked on hematoxylin/eosin sections, punched out of the paraffin block and inserted into a recipient block. A tissue arrayer (Beecher Instruments, Woodland, USA) with a core diameter of 0.6 mm was used. The RCC TMA was constructed to represent 108 cases with two spots from the tumour and two spots representing matching normal tissue from the cortex region of the kidney. In four cases, the "normal" spots did not represent kidney tissue, leaving 104 cases with matched tumour and normal tissue, plus four cases with tumour only. The whole TMA was accomplished on three paraffin blocks. Tissue spots of two tumours were lost during processing.

### Immunohistochemistry

The TMA was freshly cut (3 μm). For immunohistochemical detection of HDAC isoforms on tissue samples, prediluted polyclonal rabbit IgG antibody directed against HDAC1 (1:11, Abcam, Cambridge, UK), monoclonal mouse IgG antibody directed against HDAC2 (1:5000, Abcam) and monoclonal mouse IgG antibody directed against HDAC3 (1:500, Becton Dickinson, Franklin Lakes, NJ, USA) was used on 3 μm paraffin sections. For antigen retrieval, deparaffinized slides were placed in 0.01 M sodium citrate buffer, pH 6.0 and boiled for 5 minutes in a pressure cooker. After several rinses in TBS and pre-treatment with blocking reagent (Dako, Glostrup, Denmark) for 5 minutes, slides were incubated with primary antibody in antibody diluent solution (Zymed, San Francisco, CA, USA) for 20 minutes at room temperature and subsequently at 4°C overnight. After washing the slides in TBS, bound antibody was detected by applying a streptavidin-biotin system (BioGenex, San Ramon, CA, USA) due to a standard protocol with standard antibody dilutions as supplied by the manufacturers. For color development, a fast red system (Sigma, Deisenhofen, Germany) was used. The slides were cover slipped using Aquatex (Merck, Gernsheim, Germany).

### Evaluation of staining of tissue slides

Nuclear staining of HDAC isoforms was scored by applying a semiquantitative immunoreactivity scoring (IRS) system that incorporates the percentual area and the intensity of immunoreactivity resulting in a score ranging from 0 to 12, as described [25]. Two clinical pathologists independently scored the cases. Differences in the evaluation were discussed at a multiheaded microscope until consensus was reached. For statistical analysis, cases exhibiting an IRS from 0–6 were lumped in a HDAC *low *group whereas cases with a higher IRS (7–12) were designated HDAC *high*. This cut-off was chosen to allow for a better comparability with previous works [7-9]. Additional cut-off points (quartiles) were evaluated for each HDAC.

### Statistical analysis

Statistical analyses were performed with SPSS 15.0 (SPSS Inc., Chicago, USA). Fisher's exact and chi-square tests were applied to assess associations between expression of HDACs and clinico-pathological parameters. Correlations were computed using Spearman's bivariate rank order correlation. Univariate survival analysis was carried out according to Kaplan-Meier, differences in survival curves were assessed with the log rank test. P-values < 0.05 were considered significant.

## Results

### Expression of HDACs in renal cell cancer

**Figure 1:**
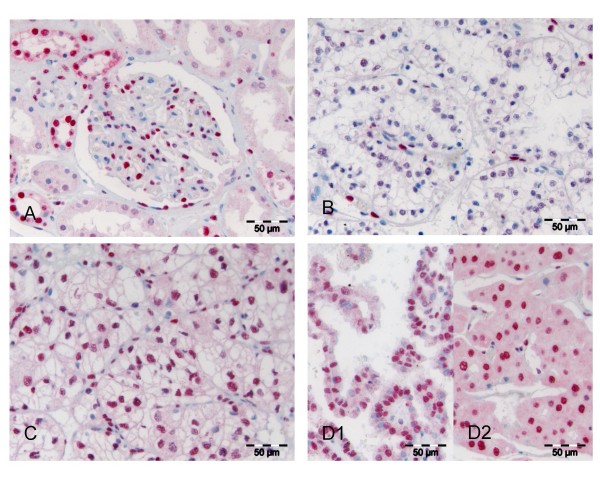
**HDAC 1 expression in normal and malignant renal tissue**. In benign renal tissue HDAC 1 is focally expressed in nuclei of mesangial cells of the glomeruli and also tubular epithelia with a stronger expression in the distal part of the nephron (**A**). Clear cell RCC negative for HDAC 1, contrasting to the relatively strong staining in adjacent stroma (**B**). RCC displaying a strong and homogenous nuclear positivity in tumour cells (**C**). Papillary (**D1**) and chromophobe (**D2**) RCC with strong HDAC 1 expression.

**Figure 2:**
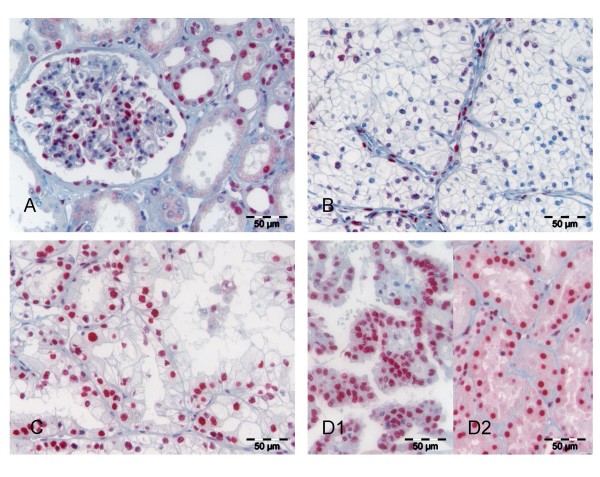
**HDAC 2 expression in normal and malignant renal tissue**. HDAC 2 was found to have a similar expression pattern as HDAC 1 with moderately positive normal glomerular and tubular cells (**A**). HDAC 2 negative clear cell carcinoma with distinctly positive stromal cells (**B**). Strong nuclear HDAC 2 expression in clear cell (**C**), papillary (**D1**) and chromophobe (**D2**) histologic subtypes of RCC.

**Figure 3:**
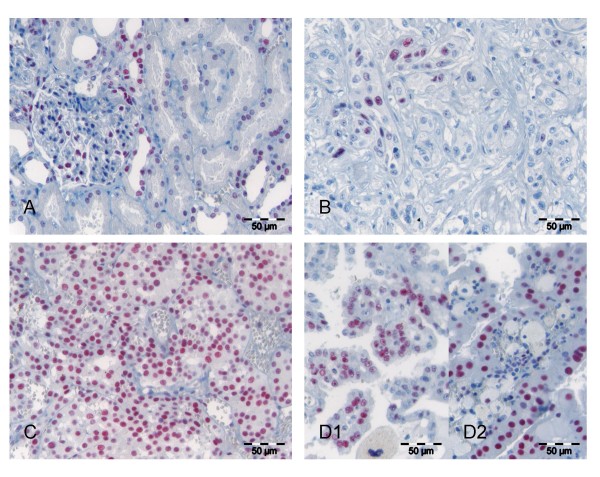
**HDAC 3 expression in normal and malignant renal tissue**. HDAC 3 expression in normal renal tissue with positive glomerular and tubular epithelial cells (**A**). Partially sarcomatoid differentiated clear cell RCC with only very few rather weak to moderately positive tumour cells (**B**). Clear cell (**C**), papillary (**D1**) and chromophobe (**D2**) RCC with strong HDAC 3 expression.

Normal renal tissue showed moderate to strong expression of all three types of class I HDACs in some but not all glomerular cells. Tubular epithelia were partially positive and expression patterns differed clearly between specific tubular subunits (Figures 1, 2, 3). It was noted that in general HDAC 3 was fainter and less frequently expressed than HDAC 1 and 2.

In renal cell carcinomas moderate to strong nuclear HDAC1, HDAC2 and HDAC3 immunoreactivity was detected in 55.7%, 56.6% and 13.2% of cases, respectively. Low or no expression of class I HDAC was seen in 44.3%, 43.4% and 86.8% for the three different isoforms whereas 9.4%, 11.3% and 72.6% of cases were completely negative (IRS 0) for the respective HDACs (Figure 1, Table 1). The low rate of positivity for HDAC 3 resulted in a low rate of cases positive (IRS>6) for all three HDACs (9.4%, n = 10), while the rate of cumulative HDAC low or negative cases (IRS ≤ 6) was 28.3% (n = 30). HDAC 1 and 2 were almost equally expressed in the different histological subtypes of renal cell cancers. In contrast, HDAC 3 was detected at high levels in only 7 (8.3%) out of 84 clear cell but in 7 of 17 (41.2%) papillary carcinomas. All five chromophobe carcinomas were negative for HDAC 3.

Interestingly, we recognized that even in HDAC negative carcinomas some intra-tumoural stromal cells expressed the class I HDACs.

### Correlation of HDAC isoform expression with clinico-pathological factors and survival

In bivariate correlation the HDAC IRS scores of all three isoforms correlated significantly with each other and with the Ki-67 (MIB-1) proliferation index (HDAC1: p = 0.009, correlation coefficient (CC): 0.252; HDAC2: p < 0.001, CC: 0.359; HDAC3: p = 0.015, CC: 0.235). Apart from a significant reciprocal correlation (Spearman rank order correlation) of HDAC3 with pT status (p = 0.005, CC -0.268) and a trend for HDAC2 in this direction (p = 0.061, CC -0.181) there were no other correlations with age, grading, nodal status, or metastasis status.

In the χ-square tests (Table 1) the above mentioned correlations could be partially confirmed in the grouped analyses. Some p values remained just above the significance level, most likely due to grouping effects.

**Figure 4:**
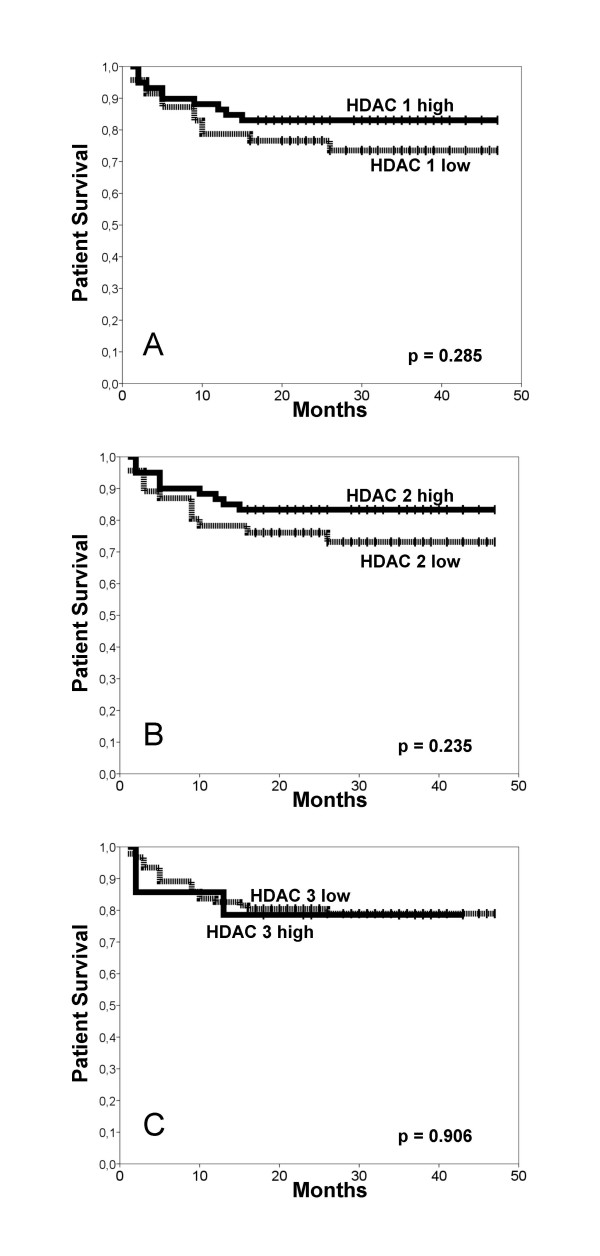
**Kaplan-Meier survival curves for renal cell cancer patients according to class I HDAC expression patterns**. For none of the HDAC isoforms 1–3 (**A-C**) a significant prognostic value for the patient survival times could be demonstrated.

Kaplan-Meyer survival analyses and log rank tests confirm the conventional prognosticators pT status, nodal status, distant metastasis and histopathological grading to be relevant for overall patient survival in our cohort (Table 2). HDAC expression did not reach significance in these analyses, neither for a single isoform (Figure 4A–C) nor in a combined analysis (not shown).

**Table 2:** Patient survival in dependence of clinico-pathological parameters and HDAC isoform expression

**Parameters**	**No. of cases**	**No. of events**	**Two Year survival time (± SE) in months**	**p-value**
**HDAC1 expression**				0.285
low	47	12	76.6 (± 6.2)	
high	59	10	83.1 (± 4.9)	

**HDAC2 expression**				0.235
low	46	12	76.1 (± 6.3)	
high	60	10	83.3 (± 4.8)	

**HDAC3 expression**				0.906
low	92	19	80.5 (± 4.1)	
high	14	3	78.6 (± 11.0)	

**Age**				0.119
≤ 61	49	7	85.7 (± 5.0)	
>61	57	15	75.4 (± 5.7)	

**pT stage**				<0.001
pT1	53	2	96.2 (± 2.6)	
pT2	3	1	66.7 (± 27.2)	
pT3	47	17	66.0 (± 6.9)	
pT4	3	2	33.3 (± 27.2)	

**Histological grade**				<0.001
G1	11	0	-	
G2	74	12	85.1 (± 4.1)	
G3	17	7	58.8 (± 11.9)	
G4	4	3	25.0 (± 21.7)	

**pN status**				0.001
pN0	50	10	80.0 (± 5.7)	
pN1/2	12	8	41.7 (± 14.2)	

**Metastasis**				<0.001
M0	47	5	89.4 (± 4.5)	
M1	23	14	43.5 (± 10.3)	

**Ki-67 index**				0.931
≤ 10%	70	15	80.0 (± 4.8)	
>10%	36	7	80.6 (± 6.6)	

All analyses were also re-computed with alterative cut-off levels, using the quartiles of the individual IRS for each HDAC. None of these tests highlighted a significant prognostic value for HDAC 1–3. Also, no correlations with clinico-pathological parameters were found. Only for pT-status there was a significant inverse association with HDAC 3 if lumped according to the quartiles (p = 0.004–0.006).

## Discussion

In this study, HDAC class I isoforms were detected in normal renal tubular and glomerular tissues and to a variable extent in renal cell cancers. The low expression rate of the HDAC3 isoform differs relevantly from that of the other two isoforms, which are highly expressed in the majority of renal tumours. These results are in contrast to the findings in all malignant tumours analyzed so far, where HDAC3 was the strongest and most frequently expressed isoform of all three class I HDACs [4,7,8]. Especially the absence of HDAC3 in the most common histological variant of RCC (clear cell RCC) suggests different regulatory mechanisms of the three isoforms in this tumour entity. However, it is interesting that there is still a significant correlation between the expression rates of all three HDACs. The connection between HDAC expression and the proliferation index (Ki-67) observed here in renal cell carcinomas has already been demonstrated for prostate cancer and colorectal cancer [8,9]. This is further in line with studies [18-20,26-28] showing that HDI treatment *in vitro *and *in vivo *leads to an arrest in tumour cell proliferation.

We and others previously reported of associations of class I HDACs with more aggressive tumours and even shortened patient survival in prostate cancer and gastric cancer [7,8,29]. In our cohort we could not find relevant and significant associations of the HDAC expression with tumour grade and other clinico-pathological parameters. This is somewhat surprising since HDACs are known to have effects on tumour cell differentiation *in vitro *and *in vivo *in other tumour entities [7-9,26,30]. However, the missing correlation of HDAC expression with tumour grade in RCC might be explained by the way tumour grade is assessed in RCC. Fuhrman grade, which is recommended by the World Health Organization, evaluates only nuclear morphology, whereas architectural features of tumour differentiation are not considered. Therefore, Fuhrman grade might be inappropriate to assess the relationship of HDAC expression to tumour differentiation, which is the predominant basis for grading schemes of other tumour entities. This might be at least a possible explanation for the missing correlations of tumor grade with HDAC expression.

Another rather unexpected finding was the reciprocal correlation of HDAC3 with tumour stage (pT-status). Given the overall low positivity for HDAC3, smaller distribution irregularities might have relevant impact on the results. In fact the papillary RCC, which were positive for HDAC3, tended to be of lower tumour stage in our cohort. If the papillary RCCs were omitted the significance of this correlation was lost.

Although high rates of HDAC expression have been found to be prognostic markers in other tumour entities, in RCC no prognostic value could be demonstrated. A shortcoming of the current study is that the acetylation status of the histones in RCC has not been assessed. Toh *et al. *have demonstrated that hyperacetylation of histones in esophageal cancer is associated with HDAC 1 overexpression which suggests that HDAC expression might be a marker of such an imbalance between acetylation and deacetylation in the neoplasm [31]. This interesting point should be focus of further study in RCC as well.

The expression of the class I HDACs in RCC might still prove useful for individual decisions whether a patient will profit from treatment with HDI, although until now it is not clear whether HDAC protein expression as assessed by immunohistochemistry is a predictor of treatment response with HDI. Clearly, additional studies are needed to clarify this point.

## Conclusion

In conclusion, we demonstrated that the class I HDAC isoforms 1 and 2 are highly expressed in the majority of renal cell cancers whereas HDAC 3 expression, in contrast to the findings in other tumour entities, could only rarely be detected, especially in clear cell RCC. Although HDAC expression in RCC was not correlated with patient survival times the expression patterns of HDACs could hypothetically be important to predict the response of RCC patients to chemotherapies comprising one of the up-coming HDAC inhibitor drugs. This should be focus of further analyses.

## Competing interests

The authors declare that they have no competing interests.

## Authors' contributions

FRF and WW coordinated the study, performed immunohistological and statistical analyses and wrote the paper. AR, VG, TB and KS performed immunohistological and statistical analyses, assisted in the cohort up-date, technical questions and wrote and revised essential parts of the paper. CS, KJ and MD provided clinico-pathological data for the study. CD supported the study coordination and provided statistical support. GK conceived and coordinated the study, performed statistical analyses, wrote and revised the paper.

## Pre-publication history

The pre-publication history for this paper can be accessed here:


